# Evolutionary Conservation and Diversification of the Translation Initiation Apparatus in Trypanosomatids

**DOI:** 10.1155/2012/813718

**Published:** 2012-07-08

**Authors:** Alexandra Zinoviev, Michal Shapira

**Affiliations:** Department of Life Sciences, Ben-Gurion University of the Negev, P.O. Box 653, Beer Sheva 84105, Israel

## Abstract

Trypanosomatids are ancient eukaryotic parasites that migrate between insect vectors and mammalian hosts, causing a range of diseases in humans and domestic animals. Trypanosomatids feature a multitude of unusual molecular features, including polycistronic transcription and subsequent processing by *trans*-splicing and polyadenylation. Regulation of protein coding genes is posttranscriptional and thus, translation regulation is fundamental for activating the developmental program of gene expression. The spliced-leader RNA is attached to all mRNAs. It contains an unusual hypermethylated cap-4 structure in its 5′ end. The cap-binding complex, eIF4F, has gone through evolutionary changes in accordance with the requirement to bind cap-4. The eIF4F components in trypanosomatids are highly diverged from their orthologs in higher eukaryotes, and their potential functions are discussed. The cap-binding activity in all eukaryotes is a target for regulation and plays a similar role in trypanosomatids. Recent studies revealed a novel eIF4E-interacting protein, involved in directing stage-specific and stress-induced translation pathways. Translation regulation during stress also follows unusual regulatory cues, as the increased translation of Hsp83 following heat stress is driven by a defined element in the 3′ UTR, unlike higher eukaryotes. Overall, the environmental switches experienced by trypanosomatids during their life cycle seem to affect their translational machinery in unique ways.

## 1. Translation Initiation in Eukaryotes

Translation is a complex process that is controlled by a large number of proteins and factors, allowing the cell to generate a rapid response to external and internal signals. The initiation step of protein synthesis is predominantly viewed as the limiting step of this process, thus serving as a target for a multitude of regulatory cues.

During cap-dependent translation initiation, a preassembled 43S preinitiation complex (PIC) is targeted to the 5′ end of the mRNA through the cap-binding complex, eIF4F. The PIC subunits are comprised of the Met-tRNA along with eIF1, eIF1A, eIF2, and the multisubunit eIF3, which is responsible for recruiting the small ribosomal subunit (reviewed in [1, 2]). The eIF4F complex consists of the cap-binding protein, eIF4E, an RNA helicase, eIF4A, and the scaffold protein, eIF4G. The mammalian eIF4G holds together the eIF4F subunits and links them to the 43S PIC by interacting with eIF4E, eIF4A, and eIF3 [3, 4]; it is also responsible for the transient circularization of translating mRNAs, by interacting with the poly(A) binding protein (PABP) that is bound at their 3′ ends (reviewed in [5, 6]). The initiation complex scans the 5′ untranslated region (5′ UTR) until it reaches the initiator AUG codon, where the 60S ribosomal subunit joins to form the mature 80S ribosome. Two factors, eIF5B [7] and eIF6 [8], participate in this step, releasing most of the initiation factors. The scanning process is assisted by helicases which are associated with the initiation complex, with eIF4A serving as the main candidate, although it is not necessarily the only factor involved in this process [9, 10]. The activity of eIF4A is enhanced by an additional factor, eIF4B, which was shown to be essential for the helicase activity [11]. Once the ribosome advances along the mRNA and vacates the AUG start codon, a new initiation complex can be formed on the 5′ cap, leading to the formation of a polyribosome. Actively translating mRNAs are usually associated with the polysomal fraction of disrupted cells.

Exposure to a variety of environmental, nutritional, or virus induced stresses, causes a global inhibition of cap-dependent translation. However, certain mRNAs continue to be translated in a cap-independent manner, by which the small ribosomal subunit is targeted to a position that is adjacent to the initiator AUG, with the aid of a highly structured RNA element in the 5′ UTR. This element is denoted the internal ribosome entry site (IRES) [12]. The IRES assigns a functional secondary structure, usually with the aid of *trans*-activating factors, such as the poly-pyrimidine-tract-binding (PTB) protein, ITAF45 or the La antigen [13]. IRES elements were also reported for endogenous mRNAs [12, 14–16], however, they do not account for all cases of cap-independent translation, and other, less understood pathways, are probably also used by eukaryotic cells [17, 18].

Translation initiation is controlled by complex signaling networks, which integrate the internal and external conditions of living cells. It was reported that the arrest in cap-dependent translation observed under stress conditions is coordinated with the dephosphorylation and activation of the 4E-binding protein (4E-BP) The latter competes with eIF4G on binding to eIF4E, thus excluding assembly of the cap-binding complex [19–21]. The phosphorylation status of 4E-BP is mediated by the TOR-based complexes [22] that link between metabolism, protein synthesis and cell cycle [23, 24], which are controlled by a variety of cellular pathways [25]. While in most cases, blocking the dephosphorylation of 4E-BP led to inhibition of protein synthesis, exceptional cases have been reported where protein synthesis was not interrupted despite the dephosphorylation of 4E-BP, which was induced by specific mTOR inhibitors [26].

Another way by which translation can be globally stopped is through phosphorylation of eIF2*α*, which forms a ternary complex with Met-tRNA-GTP. Following the hydrolysis of GTP, activity of eIF2*α* is regenerated by the eIF2B-mediated exchange of GDP with GTP. It is commonly observed that phosphorylation of eIF2*α* at Ser 51 blocks this exchange, resulting in a global translational arrest [27].

Translation can be regulated also at a gene-specific level, without affecting the overall translation capacity of the cell. In many cases, a regulatory protein that is recruited by elements in the 3′ UTR binds eIF4E, in a manner that blocks the access to eIF4G and prevents assembly of the eIF4F complex. Such a pattern of regulation is observed during the development of *Drosophila* embryos, in which specific proteins and mRNAs are distributed unevenly along the body axis. For example, Maskin interacts with the eIF4E only on RNAs that contain a cytoplasmic polyadenylation element (CPE); disruption of the eIF4E-eIF4G complex by this protein is therefore specific to mRNAs that contain a CPE in their 3′ UTR (reviewed in [21]).

Exposure to extreme conditions such as temperature and pH alterations, osmotic switches, UV irradiation, nutrient starvation and oxidative stress is hazardous to all living cells, and is combatted by activation of a complex chaperone network. Extreme temperatures cause cellular damage at multiple levels, accompanied by a global arrest in synthesis of most cellular proteins, except for heat shock proteins (HSPs). In cases where the cell cannot overcome the inflicted damage, apoptosis may be induced [16]. The increased translation of HSPs in higher eukaryotes is controlled by the 5′ UTR, as shown for both HSP70 [28, 29] and HSP90 [30]. HSP90 and some variants of HSP70 are expressed at ambient temperatures, but their translation increases dramatically during heat shock. Despite extensive efforts, no functional motifs that could drive preferential translation at elevated temperatures were yet identified. It was also suggested that under normal temperatures, translation of Hsp90 occurs by a typical cap-dependent scanning mechanism, whereas during heat shock, translation shifts to a cap-independent mode.

## 2. Trypanosomatid Organisms

Trypanosomatids are unicellular, diploid protists from the Kinetoplastidæ order, which migrate between insect vectors and mammalian hosts, causing a variety of parasitic diseases in humans and their domestic animals.* Leishmania* parasites reside in the alimentary tract of female sandflies as extracellular flagellated promastigotes [31, 32]. Upon transmission to mammalian hosts, they differentiate into obligatory intracellular amastigotes within macrophages and dendritic cells [33, 34], causing a range of symptoms, that depend on the infecting species. *Trypanosoma cruzi* is the causative agent of Chagas disease in South America, and *T. brucei *spp. cause the African sleeping sickness. Altogether, trypanosomatid-borne diseases cause a great health threat and economical drawbacks for native populations living in endemic countries, as well as being a major problem for travelers visiting these regions. The divergence of most trypanosomatid species occurred very early in evolution, before the emergence of both vectors and hosts [35]. Thus, unique molecular properties are common in this family as compared to higher eukaryotes.

**Figure 1:**
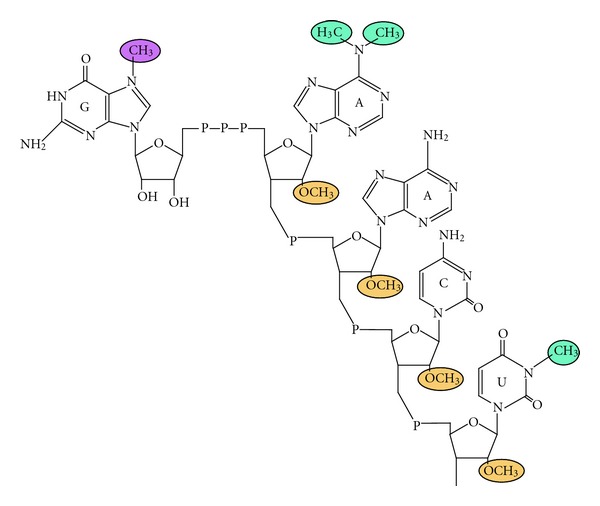
Trypanosomatid cap-4 structure, based on Bangs et al., 1992 [36].

 Protein coding genes in trypanosomatids are arranged as large, unidirectional transcription units, which are transcribed polycistronically. The resulting pre-mRNAs are matured by *trans*-splicing and polyadenylation into mono-cistronic mRNAs (reviewed in [37, 38]). Conventional RNA polymerase II promoters were not identified to date, and there is yet no evidence for the occurrence of regulated transcription activation processes for protein coding genes. Thus, mRNA processing, stability and translation serve as key mechanisms that direct differential program of gene expression throughout the pirasite life cycle (reviewed in [39]). During *trans*-splicing, a conserved mini-exon of 39 nucleotides is donated by the spliced leader RNA (SL RNA) to the 5′ end of all mRNAs. This mini-exon provides the 5′ cap structure, which in trypanosomatids is heavily methylated on the first four nucleotides and is thus denoted cap-4 [40]. In addition to the consensus m^7^GTP, cap-4 consists of 2′-*O* ribose methylations on the first four nucleotides of the SL RNA and two base methylations on the first adenosine and fourth uridine (Figure 1). These base methylations are unique to trypanosomatids, and are not known in any other group of eukaryotes [36]. Substitutions of individual nucleotides in the cap-4 structure diminished the ability of the mutated SL RNA to be utilized in *trans-*splicing reactions [41] and thus it was not possible to evaluate the exact role of ribose methylation in translation. The enzymes that promote the 2′-*O*-methylations on the ribose moieties of the cap-4 nucleotides were identified [42, 43], and the effect of their elimination was tested. Single and double knock-out mutants of genes encoding one or two of the enzymes responsible for ribose 2′-*O*-methylation at position 1 (TbMTr1), 2 (TbMTr2), 3 and 4 (TbMTr3) were examined. Preventing the ribose methylations at positions 3 and 4, but not at position 1, reduced the translation rates; a further exacerbation was observed by additional loss of the methylation at position 2. Knock-out of TbMTr1 alone did not cause an inhibitory effect on translation, however, depletion of TbMTr2 or TbMTr3 on TbMTr1^−/−^ background did not yield viable parasites [44, 45]. Therefore, it was proposed that only a minimal level of mRNA cap ribose methylation is essential for trypanosome viability. The role of cap-1 modification was shown to be related to the SL RNA biogenesis, as formerly shown for *Leptomonas collosoma* mutants [38].

## 3. eIF4E Isoforms of Trypanosomatids

Trypanosomatid genome annotation combined with a functional analysis approach, paved the way for studies of the translation apparatus in these organisms. Some of the factors were subject to a thorough biochemical and cellular analysis, whereas others were only identified based on sequence homology. Homology modeling of the four LeishIF4E paralogs suggested that the structure of the cap-binding pocket was conserved and maintained the basic features observed for the yeast and mammalian eIF4E [46]. It is of interest to note that the association constants for complexes of LeishIF4E homologues with m^7^GTP as well as with cap-4 were two orders of magnitude lower than those of the mouse protein [46]. This could be related to the evolutionary changes that occurred in the parasite proteins to promote their interaction with cap-4. However, this has yet to be proven.

eIF4E is the eukaryote 5′ cap-binding translation initiation factor; its association with eIF4G is fundamental to the assembly of the cap-binding complex. The 3D structure of eIF4E from several organisms was formerly deciphered [3, 47–50]. Its N-terminal part is flexible and not conserved, while the C-terminus adopts a conserved structural signature. eIF4E acquires the shape of a baseball glove with a dedicated pocket for binding of the methylated guanine, which contains a sandwich of three Trp residues (W56, 102 and 166, according to the murine numbering). In addition, the basic residues Arg112, 157 and 162 interact with the negatively charged phosphate moieties of the cap structure. eIF4E binds eIF4G and several translation repressors, such as 4E-BP, through several conserved residues in its C-terminus. The high degree of structural conservation among orthologs of eIF4E enables homologs from different species to functionally replace each other in a yeast-based genetic complementation assay [51].

The trypanosomatid cap-4 binding proteins have gone through structural adaptations during their evolution to adjust to binding of the highly modified cap-4 structure. Their genomes encode four isoforms of eIF4E [46, 52]. Although homology modeling of the four proteins supports a structural conservation of the cap-binding pocket, none of the four paralogs could complement the missing function of the yeast eIF4E, indicating high functional divergence from their higher eukaryotic orthologs [46]. Thus, deciphering the roles of the different isoforms is complex, especially since efficient cell-free systems for *in vitro* reconstitution of translation initiation using parasite mRNAs are not available for trypanosomatids. Biophysical assays using a chemically synthesized cap-4 and intermediate cap analogues [53], showed that the four eIF4E, denoted LeishIF4E-1 through LeishIF4E-4, vary in their cap-binding specificities. Trp fluorescence assays indicated that LeishIF4E-1 and LeishIF4E-4 bound m^7^GTP and cap-4 with comparable affinities, LeishIF4E-3 bound mainly to m^7^GTP and hardly to cap-4, whereas LeishIF4E-2 showed a great preference to cap-4, as compared to m^7^GTP [46]. In agreement, the endogenous LeishIF4E-1 and LeishIF4E-4 were eluted from m^7^GTP-Sepharose column [46, 54]. Despite the ability of LeishIF4E-3 to bind m^7^GTP in the fluorescence titration assays, the recombinant and endogenous proteins showed very weak binding to m^7^GTP-Sepharose column, for reasons not fully understood yet [46].

The subunits of the *Leishmania *eIF4F complex were examined by pulldown analysis and by monitoring their migration profile on sucrose gradients. These analyses led to the conclusion that LeishIF4E-4 is the most probable candidate to serve as the conventional eIF4E in promastigotes, and is part of the parasite LeishIF4F cap-4 binding complex. LeishIF4E-4 was shown to interact with LeishIF4G-3, a protein that contains a “middle of eIF4G” (MIF4G) domain [54, 55], which is responsible for recruiting LeishIF4A-1. A parallel interaction was shown for the *Trypanosoma brucei* ortholog [54]. The three eIF4F subunits comigrate on sucrose density gradients and are found in fractions that are expected to contain the pre-initiation complex [55].

Silencing experiments by RNAi were performed on the four *T. brucei *isoforms [54] in an attempt to reveal their function. Downregulation of TbIF4E-4 inhibited the growth of bloodstream form, but not procyclic cells. This result does not coincide with data obtained for the *Leishmania *paralog, since LeishIF4E-4 binds m^7^GTP and LeishIF4G-3 mainly in promastigotes, and fails to perform these activities upon exposure to mammalian-like temperatures and in axenic amastigotes [56]. Furthermore, the migration pattern of LeishIF4E-4 in sucrose gradients shows that in heat-shocked cells this protein no longer forms large complexes. Altogether, the activity of LeishIF4E-4 is dramatically reduced at prolonged elevated temperatures [55], through a mechanism which is yet to be resolved. The differences observed between the stage-specific function of the LeishIF4E-4 and TbIF4E-4 described above could originate from a variable experimental setup, or alternatively, from differences between the species. It is also possible that after the final differentiation to the mammalian amastigote life-form, LeishlF4E-4 resumes its activity.

The dual silencing of TbIF4E-4 and TbIF4E-1 led to a growth arrest of procyclic parasites, suggesting that the two proteins may have a partially redundant function. Individual silencing of TbIF4E-4 or TbIF4E-1 was harmful only to the bloodstream life form. LeishIF4E-1, the *Leishmania* ortholog of TbIF4E-1, is the only eIF4E isoform that maintains its expression at elevated temperatures and in axenic amastigotes of *Leishmania*. The other three orthologs were downregulated under these conditions, excluding that they have a role in translation during heat shock [46, 56]. It is, therefore, possible that LeishIF4E-1 is associated with translation under stress and in amastigotes. TbIF4E-1, as well as its *Leishmania* ortholog LeishIF4E-1, do not interact efficiently with TbIF4G-3 or LeishIF4G-3, respectively [54, 56], suggesting that this isoform does not participate in building an eIF4F complex and therefore, could promote alternative pathways of translation, possibly in an eIF4G-independent manner. LeishIF4E-1 could either have an active role in translation which has not yet been resolved, or alternatively, it could passively protect the cap structure, if cap-independent mechanisms are practiced.

Elimination of TbIF4E-2, had no effect on *T. brucei* growth in both life forms [54]. The *Leishmana*, LeishIF4E-2, ortholog comigrated with heavy polysomes in sucrose gradients [46], unlike typical translation initiation factors that are found in lighter fractions [57, 58]. The RNAi experiments in *T. brucei* indicated that among the four paralogs, only TbIF4E-3 was essential for growth of both procyclic and bloodstream life forms. Its silencing caused a reduction in incorporation of a radiolabeled amino acid into newly synthesized polypeptides, and it was, therefore, suggested that TbIF4E-3 serves as a translation initiation factor [54]. However, the reduced translation rates occurred only after 72 hours, while the silencing was observed already after 24 hours. It could therefore be a downstream effect of other processes that inhibit cell growth in the absence of TbIF4E-3. TbIF4E-3 was reported to be part of RNA granules in *T. brucei *[59, 60] and could, therefore, be associated with trafficking of mRNAs either to storage bodies or to actively translating polysomes. The low affinity of the *Leishmania *ortholog LeishIF4E-3 to cap-4 contradicts its definition as a typical translation initiation factor. Thus, although TbIF4E-3 interacts with a MIF4G domain protein, TbIF4G-4, its precise role remains elusive.

## 4. eIF4E Binding Proteins of Trypanosomatids

Trypanosomatids encode several eIF4G candidates that contain the MIF4G domain. This element consists of several HEAT repeats [61], typical of all eIF4G proteins in higher eukaryotes [62]; it is responsible for binding to eIF4A as well as to RNA. The C-terminus of the human eIF4G contains a second site for interaction with eIF4A and its N-terminus carries binding sites for eIF4E and PABP. A consensus peptide motif, YXXXXLΦ, in eIF4G and in 4E-BP is responsible for binding to eIF4E. Substitution of the conserved Y or LΦ residues in the motif abrogates the eIF4E-eIF4G interaction [63].

The identity of the *Leishmania* eIF4G ortholog was deduced by its co-purification and interaction with LeishIF4E-4. This binding was monitored in yeast two-hybrid assays and pulldown experiments [52, 54–56]. LeishIF4G-3 and TbIF4G-3 contain a typical MIF4G domain [52, 55], but they vary from their mammalian counterpart in other parts of the protein. For example, the N-terminus of LeishIF4G-3 is short (50 amino acids) and contains the LeishIF4E-4 binding-peptide, but it cannot interact with PABP in a yeast two-hybrid assay. This peptide motif is only partially conserved with the YXXXXLΦ consensus, and binding to LeishIF4E-4 requires the presence of a Phe residue at position 4 [63], in addition to Tyr and Leu at positions 1 and 6 of the peptide. A partial requirement was observed for the Gly and Glu residues at positions 3 and 8 [55]. Altogether, this indicates a certain degree of variability from the eukaryote consensus sequence.

**Figure 2:**
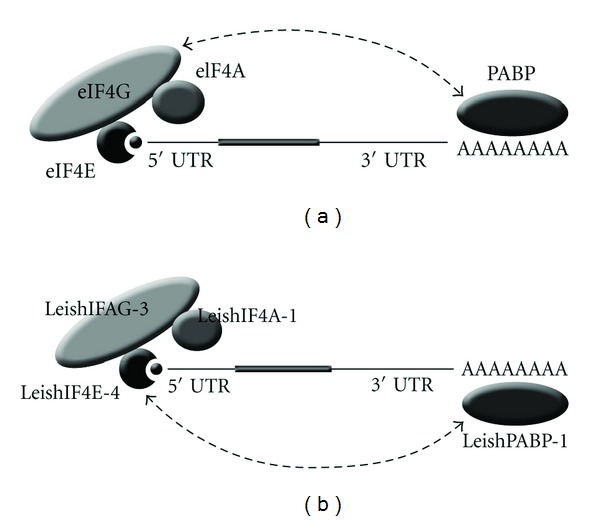
Interacting partners of cap-binding proteins in *Leishmania*. The typical eIF4F complex of higher eukaryotes (a) and *Leishmania* promastigotes (b) are shown. A table summarizing the known interacting partners of the *Leishmania* eIF4F complex are shown in Table 1. Accession numbers of the described proteins are: LeishIF4E-1—LmjF27.1620, LeishIF4E-3—LmjF28.2500, LeishIF4E-4—LmjF30.0450, LeishIF4G-3—LmjF16.1600, LeishIF4G-4—LmjF36.6060, LeishIF4A-1—LmjF01.0780 and LmjF01.0770, LeishPABP-1—LmjF35.5040, Leish4E-IP—LmjF35.3980.

 Other regions of the parasite eIF4G are also a source for variability, as compared to its human counterpart. eIF4G is a scaffold protein that links the 5′ and 3′ ends by interacting with both eIF4E and PABP. However, unlike in higher eukaryotes, mRNA circularization occurs through an interaction between the N-terminus of LeishIF4E-4 and LeishPABP-1. Elimination of this LeishIF4E-4 domain prevents its binding to LeishPABP-1 [56]. Therefore, LeishIF4E-4 along with LeishIF4G-3 and LeishIF4A-1, comprise a typical eIF4F complex in promastigotes [64], see Figure 2. The binding between LeishIF4G-3 and LeishIF4E-4 is eliminated at elevated temperatures and in axenic amastigotes, supporting that under these conditions translation may proceed through alternative pathways, possibly involving LeishIF4E-1.

 An additional eIF4E-eIF4G pairing was reported for TbIF4E-3 and TbIF4G-4 [54]. The authors of this study propose that TbIF4E-3 is involved in translation initiation. This assumption is not supported by studies in *Leishmania* that excluded the copurification of these two proteins over a m^7^GTP affinity column [54]. Furthermore, LeishIF4E-3 failed to bind cap-4 in fluorescence titration assays, thus making it an unlikely candidate to serve as a conventional translation initiation factor [46].

Another well-known eIF4E binding protein is 4E-BP of higher eukaryotes. It is a highly conserved protein that is expressed in most eukaryotes, except for *C. elegans*. The trypanosomatid genome database also does not contain any ortholog of the consensus 4E-BP (~10 kDa). However, affinity co-purification assays identified a novel LeishIF4E-interacting protein, denoted Leish4E-IP (~80 kDa) [56]. Leish4E-IP and the eukaryotic 4E-BP show no sequence homology, but share a predicted unstructured nature. Leish4E-IP binds mainly to LeishIF4E-1 with a tight requirement for the consensus YXXXXLΦ peptide [63]. Furthermore, although Leish4E-IP is expressed at all life stages, its binding to LeishIF4E-1 is observed only in promastigotes, suggesting that it participates in framing a stage-specific program of gene expression, via a mechanism that is yet to be resolved. Posttranslational modifications, such as phosphorylations, are likely to be involved. Data mining in the *Leishmania* genome revealed three orthologs of the TOR kinase. TOR 1 and 2 are essential, as null mutants could not be generated, whereas TOR3 could be eliminated. However, null mutants of TOR3 were nonvirulent and were impaired in their ability to survive within macrophages [65].

## 5. eIF4A Isoforms of Trypanosomatids

eIF4A is a member of the DEAD-box RNA helicases [66]. It promotes scanning of the 5′ UTR by the 43S PIC, via unwinding of the secondary RNA structures, until it arrives at the initiator AUG codon. DEAD-box proteins participate in a multitude of processes related to transcription, RNA processing, export and translation. The genome of most eukaryotes encodes for more than a single eIF4A isoform. In addition to the translation initiation factor eIF4A, another paralog was reported to be part of the exon junction complex (EJC) [67]. The genome of *T. brucei *encodes two isoforms of eIF4A. TbIF4AI and its *Leishmania* homolog, LeishIF4A-1, are part of the eIF4F cap-binding complex. Tb4AI is cytoplasmic and, as expected, interacts with TbIF4G-3 [52, 56]. Its downregulation caused a dramatic decrease in protein synthesis. TbIF4AIII is predominantly nuclear. It is essential, but its silencing hardly affects protein synthesis, and the resulting lethal effects are delayed, as compared to TbIF4AI. Thus TbIF4AI is the translation initiation factor that comprises the cap-4 binding complex, while the role of TbIF4AIII is not yet understood, although it may assign a similar role as in higher eukaryotes [64].

## 6. Poly(A) Binding Proteins of Trypanosomatids

Yeast encode a single PABP, whereas metazoans express several paralogs, with a tissue, or embryonic-specific pattern of expression [68]. The typical PABP are closely related, and consist of four conserved RRM domains. Their C-terminus promotes protein-protein interactions with their binding partners [69].

Elongated poly(A) tails are crucial for translation, but are also involved in a variety of processes related to RNA processing, export and stabilization. The mammalian PABP associates with eIF4G, resulting in transient circularization of the mRNA during translation initiation [70]. This interaction stabilizes the initiation complex and enhances translation. It is therefore targeted by endogenous regulators, such as the PABP-binding proteins PAIP1 and PAIP2, which can enhance or repress the activity of PABP, respectively [71]. PABP is also targeted by viral proteases, as part of their strategy to take over the cellular translational machinery [72].

*T. brucei* and *T. cruzi* genomes contain two PABP paralogs [73, 74]. Both are essential, indicating that they have different cellular functions. The leishmanias encode another unique isoform, PABP3 [73]. PABP1 was associated with components of the cap-binding complex [56, 73], suggesting that it is the cap-dependent translation initiation factor. To date, it is the only isoform that was shown to bind directly to a translation initiation factor. However, unlike in higher eukaryotes, it interacts with eIF4E instead of eIF4G. The higher eukaryote PABP shuttles between the nucleus and the cytoplasm, but it is mostly cytoplasmic. All three *Leishmania* isoforms are indeed cytoplasmic, but inhibition of transcription causes PABP2 and PABP3 to accumulate in the nucleus. Their role is yet to be identified.

## 7. Translation under Stress Conditions in Trypanosomatids

The untranslated regions of trypanosomatid mRNAs, mostly those located downstream to the coding sequences, play a key role in differential expression of genes during the life cycle. This was established by the use of reporter systems for Hsp70 [75, 76] and Hsp83 [77, 78] of *Leishmania*. It was also established, that despite the key role of mRNA stability, elements that affected translation of Hsp83 alone could be identified. Large mutations that destabilized the Hsp83 mRNA at elevated temperatures did not interfere with its preferential translation, as long as the regulatory element for translation was included in the 3′ UTR [77]. Fine deletions finally identified a regulatory element of 30 nucleotides (positions 312–341), containing a stretch of polypyrimidines. This region was shown to be part of an RNA structure that was predicted with high probability [79], using the UNAfold algorithm [80]. A biophysical evaluation of the mRNA melting curves was performed to examine the role of secondary structures in the regulatory region. Incubation of the corresponding wild-type mRNA fragment (1–472) led to its melting at elevated temperatures (35°C). The mutant element, that did not induce preferential translation, failed to show a similar pattern. It was, therefore, assumed that preferential translation of *Leishmania *HSP83 during stress was promoted by melting of the regulatory region in the mRNA [79]. It is interesting to note that the element is not conserved throughout trypanosomatids, emphasizing the role of RNA structure in this mode of regulation.

An additional RNA element that confers stage-specific translation was identified in the the *Leishmania* Amastin genes. It was first mapped as an element of 450 nucleotides within their 3′ UTRs. Additional experiments narrowed this region down to 150 nucleotides that enhance translation of the harboring transcript in the amastigote life stage. The Amastin element was found in other amastigote-specific genes of *Leishmania*, such as Hsp100 [81, 82]. However, it bears no conservation to that of Hsp83. Furthermore, no defined secondary structure was reported for the Amastin RNA regulatory sequence. Therefore, the preferential translation conferred by the two elements is functionally distinct.

Trypanosomatids experience a broad range of environmental stresses during their complex life cycle. In addition to the switch between vector and host, they can also suffer from a shortage in sugars within the insect vector, due to its nutritional diet. Under these conditions, a transient arrest of translation is required until the stress is relieved. One common way to arrest global translation in higher eukaryotes is through inactivation of eIF2*α*, which can be achieved by phosphorylation of its conserved Ser51 residue. Indeed, phosphorylation of the *T. brucei* paralog occurs on the corresponding Thr169 [83] and on Thr 166 in the *Leishmania* eIF2*α* [84]. Since the trypanosomatid ortholog has a long non-conserved N-terminal extension, it shifts the position of the phosphorylated Thr. This phosphorylation of the *Leishmania* eIF2*α* is associated with stage transformation [84]. However, a mutant of *T. brucei* strain that encodes for a mutated eIF2*α* (T169A/−) and therefore cannot undergo phosphorylation, showed no effect on translation or on the ability to form heat-shock stress granules [59]. Thus, phosphorylation of Thr169 alone is most probably not sufficient to inhibit translation. Three potential eIF2*α* kinases (TbeIF2K1 to -3) were identified in the genome of *T. brucei* [83]. TbeIF2K2 was extensively studied, and shown to localize in the membrane of the flagellar pocket, a site that is known to promote exo- and endocytosis. This kinase phosphorylates the trypanosomatid eIF2*α*, as well as its mammalian counterpart. However, TbeIF2*α* is not a substrate for the mammalian GCN2 or PKR.

A second conserved mechanism for global translational arrest in higher eukaryotes involves the dephosphorylation of 4E-BP, although recent reports show numerous exceptions [26], indicating that this pathway is much more complex [23]. In most cases, the dephosphorylated 4E-BP binds to eIF4E and prevents assembly of the cap-dependent translation initiation complex. Trypanosomatid genomes lack the consensus 4E-BP and this pathway to achieve translational arrest is not functional as well. Thus, the mechanisms that confer a global decrease in translation in trypanosomatids remain unclear.

Differentiation of trypanosomatids from the insect-specific to the mammalian life-form is induced by extreme environmental switches. This raises the question of how these parasites deal with the damaging effects of the prolonged stresses, which would be deadly to other eukaryotes. In the absence of conventional mechanisms for transcriptional activation of protein coding genes [37], their differential pattern of expression along the life cycle is induced by post-transcriptional regulatory mechanisms [39], including splicing and mRNA stability, as well as translation [76, 77, 85]. Transcriptome [86, 87] and proteome [88] screens performed with *Leishmania *parasites during their axenic differentiation were published and are publicly available. In total, they reveal that differential gene expression occurs due to changes in mRNA levels at the early stages of differentiation. However, at later stages, translation and posttranslational regulation mechanisms are more influential [84]. It was also noted by the Zilberstein group that during the initial period at which signaling for differentiation takes place, translation decreased dramatically. Expression of ribosomal proteins was downregulated, eIF2*α* was phosphorylated and a general decrease in the amount of polysomes was observed. These effects were mainly transient, and translation resumed upon completion of the differentiation process [88].

## 8. What Is Next?

The research of trypanosomatids is rapidly advancing, and new tools are constantly being developed. The use of RNAi, which currently is restricted to *T. brucei*, is about to be developed for *Leishmania braziliensis* [88], the only *Leishmania* species that encodes all the components of this pathway. It should be interesting to see if silencing of the various factors described in this review confers similar effects in *Leishmania* as compared to *Trypanosoma*.

Another interesting point refers to the elegant strategy developed by *Leishmania* parasites to overtake their host cells. It was recently shown that the GP63 protease, which is secreted by the parasites into the infected macrophage, causes an efficient shutdown of the host protein synthesis. This is achieved by cleavage of mTOR, causing the constitutive dephosphorylation and activation of 4E-BP [89]. However, other targets of mTOR could presumably be affected, resulting in inhibitory effects on the macrophage metabolism.

Several recent publications describe the translational machinery of trypanosomatids and highlight its evolutionary variability, as compared to higher eukaryotes. The reported diversifications provide an exciting target for novel therapeutic approaches [90]. Potential drug targets that could be pursued include protein-RNA and protein-protein interactions that promote assembly of the translation initiation complex. For example, interactions between the cap-binding proteins and the unique cap-4 structure are of interest, as well as the binding between LeishIF4E-4 and LeishIF4G-3. TbIF4E-3, the ortholog of LeishIF4E-3, is also an interesting drug target, as it was shown to be essential in the blood stream form of *T. brucei.*

## Figures and Tables

**Table 1:** 

Protein	Interacts with	Source
LeishIF4E-1	Leish4E-IP	Zinoviev et al., 2011 [56]
LeishIF4E-3	LeishIF4G-4	Freire et al., 2011 [54]
LeishIF4E-4	LeishIF4G-3	Yoffe et al., 2009 [55]
LeishIF4A-1	LeishIF4G-3	Zinoviev et al., 2011 [56]
LeishPABP-1	LeishIF4G-3	Zinoviev et al., 2011 [56]

## References

[1] Gingras AC, Raught B, Sonenberg N (1999). eIF4 initiation factors: effectors of mRNA recruitment to ribosomes and regulators of translation. *Annual Review of Biochemistry*.

[2] Pestova TV, Lorch JR, Hellen CHT, Mathews MB, Sonenberg N, Hershey JWB (2007). The mechanism of translation initiation in Eukaryotes. *Translation Control in Biology and Medicine*.

[3] Gross JD, Moerke NJ, Von Der Haar T (2003). Ribosome loading onto the mRNA cap is driven by conformational coupling between eIF4G and eIF4E. *Cell*.

[4] Yamamoto Y, Singh CR, Marintchev A (2005). The eukaryotic initiation factor (eIF) 5 HEAT domain mediates multifactor assembly and scanning with distinct to interacts to eIF1, eIF2, eIF3, and eIF4G. *Proceedings of the National Academy of Sciences of the United States of America*.

[5] Amrani N, Ghosh S, Mangus DA, Jacobson A (2008). Translation factors promote the formation of two states of the closed-loop mRNP. *Nature*.

[6] Cheng S, Gallie DR (2007). eIF4G, eIFiso4G, and eIF4B bind the poly(A)-binding protein through overlapping sites within the RNA recognition motif domains. *Journal of Biological Chemistry*.

[7] Unbehaun A, Marintchev A, Lomakin IB (2007). Position of eukaryotic initiation factor eIF5B on the 80S ribosome mapped by directed hydroxyl radical probing. *EMBO Journal*.

[8] Gandin V, Miluzio A, Barbieri AM (2008). Eukaryotic initiation factor 6 is rate-limiting in translation, growth and transformation. *Nature*.

[9] Oberer M, Marintchev A, Wagner G (2005). Structural basis for the enhancement of eIF4A helicase activity by eIF4G. *Genes and Development*.

[10] Imataka H, Sonenberg N (1997). Human eukaryotic translation initiation factor 4G (eIF4G) possesses two separate and independent binding sites for eIF4A. *Molecular and Cellular Biology*.

[11] Methot N, Pause A, Hershey JWB, Sonenberg N (1994). The translation initiation factor eIF-4B contains an RNA-binding region that is distinct and independent from its ribonucleoprotein consensus sequence. *Molecular and Cellular Biology*.

[12] Hellen CUT, Sarnow P (2001). Internal ribosome entry sites in eukaryotic mRNA molecules. *Genes and Development*.

[13] Pilipenko EV, Pestova TV, Kolupaeva VG (2000). A cell cycle-dependent protein serves as a template-specific translation initiation factor. *Genes and Development*.

[14] Bert AG, Grépin R, Vadas MA, Goodall GJ (2006). Assessing IRES activity in the HIF-1*α* and other cellular 5′ UTRs. *RNA*.

[15] Holcik M, Sonenberg N, Korneluk RG (2000). Internal ribosome initiation of translation and the control of cell death. *Trends in Genetics*.

[16] Holcik M, Sonenberg N (2005). Translational control in stress and apoptosis. *Nature Reviews Molecular Cell Biology*.

[17] Andreev DE, Dmitriev SE, Terenin IM, Prassolov VS, Merrick WC, Shatsky IN (2009). Differential contribution of the m^7^G-cap to the 5′
end-dependent translation initiation of mammalian mRNAs. *Nucleic Acids Research*.

[18] Mitchell SF, Walker SE, Algire MA, Park EH, Hinnebusch AG, Lorsch JR (2010). The 5’-7-methylguanosine cap on eukaryotic mRNAs serves both to stimulate canonical translation initiation and to block an alternative pathway. *Molecular Cell*.

[19] Pause A, Belsham GJ, Gingras AC (1994). Insulin-dependent stimulation of protein synthesis by phosphorylation of a regulator of 5’-cap function. *Nature*.

[20] Lin TA, Kong X, Haystead TAJ (1994). PHAS-I as a link between mitogen-activated protein kinase and translation initiation. *Science*.

[21] Richter JD, Sonenberg N (2005). Regulation of cap-dependent translation by eIF4E inhibitory proteins. *Nature*.

[22] Lawrence JC, Abraham RT (1997). PHAS/4E-BPs as regulators of mRNA translation and cell proliferation. *Trends in Biochemical Sciences*.

[23] Laplante M, Sabatini DM (2009). mTOR signaling at a glance. *Journal of Cell Science*.

[24] Gingras AC, Gygi SP, Raught B (1999). Regulation of 4E-BP1 phosphorylation: a novel two step mechanism. *Genes and Development*.

[25] Laplante M, Sabatini DM (2009). An emerging role of mTOR in lipid biosynthesis. *Current Biology*.

[26] Feldman ME, Apsel B, Uotila A (2009). Active-site inhibitors of mTOR target rapamycin-resistant outputs of mTORC1 and mTORC2. *PLoS Biology*.

[27] Dever TE (2002). Gene-specific regulation by general translation factors. *Cell*.

[28] McGarry TJ, Lindquist S (1985). The preferential translation of Drosophila hsp70 mRNA requires sequences in the untranslated leader. *Cell*.

[29] Klemenz R, Hultmark D, Gehring WJ (1985). Selective translation of heat shock mRNA in Drosophila melanogaster depends on sequence information in the leader. *EMBO Journal*.

[30] Ahmed R, Duncan RF (2004). Translational regulation of Hsp90 mRNA: AUG-proximal 5′-untranslated region elements essential for preferential heat shock translation. *Journal of Biological Chemistry*.

[31] Sacks DL, Perkins PV (1984). Identification of an infective stage of Leishmania promastigotes. *Science*.

[32] Pimenta PFP, Turco SJ, McConville MJ, Lawyer PG, Perkins PV, Sacks DL (1992). Stage-specific adhesion of Leishmania promastigotes to the sandfly midgut. *Science*.

[33] Chang KP, Dwyer DM (1976). Multiplication of a human parasite (Leishmania donovani) in phagolysosomes of hamster macrophages *in vitro*. *Science*.

[34] Chang KP, Fong D, Bray RS, Chang KP, Bray RS (1985). Biology of Leishmania and leishmaniasis. *Leishmaniasis*.

[35] Fernandes AP, Nelson K, Beverley SM (1993). Evolution of nuclear ribosomal RNAs in kinetoplastid protozoa: perspectives on the age and origins of parasitism. *Proceedings of the National Academy of Sciences of the United States of America*.

[36] Bangs JD, Crain PF, Hashizume T, McCloskey JA, Boothroyd JC (1992). Mass spectrometry of mRNA cap 4 from trypanosomatids reveals two novel nucleosides. *Journal of Biological Chemistry*.

[37] Clayton CE (2002). Life without transcriptional control? From fly to man and back again. *EMBO Journal*.

[38] Michaeli S (2011). Trans-splicing in trypanosomes: machinery and its impact on the parasite transcriptome. *Future Microbiology*.

[39] Clayton C, Shapira M (2007). Post-transcriptional regulation of gene expression in trypanosomes and leishmanias. *Molecular and Biochemical Parasitology*.

[40] Liang XH, Haritan A, Uliel S, Michaeli S (2003). trans and cis splicing in trypanosomatids: mechanism, factors, and regulation. *Eukaryotic Cell*.

[41] Mandelboim M, Estraño CL, Tschudi C, Ullu E, Michaeli S (2002). On the role of exon and intron sequences in trans-splicing utilization and cap 4 modification of the trypanosomatid Leptomonas collosoma SL RNA. *Journal of Biological Chemistry*.

[42] Ruan JP, Ullu E, Tschudi C (2007). Characterization of the Trypanosoma brucei cap hypermethylase Tgs1. *Molecular and Biochemical Parasitology*.

[43] Arhin GK, Ullu E, Tschudi C (2006). 2′
-O-Methylation of position 2 of the trypanosome spliced leader cap 4 is mediated by a 48 kDa protein related to vaccinia virus VP39. *Molecular and Biochemical Parasitology*.

[44] Zamudio JR, Mittra B, Campbell DA, Sturm NR (2009). Hypermethylated cap 4 maximizes Trypanosoma brucei translation. *Molecular Microbiology*.

[45] Zamudio JR, Mittra B, Zeiner GM (2006). Complete cap 4 formation is not required for viability in Trypanosoma brucei. *Eukaryotic Cell*.

[46] Yoffe Y, Zuberek J, Lerer A (2006). Binding specificities and potential roles of isoforms of eukaryotic initiation factor 4E in Leishmania. *Eukaryotic Cell*.

[47] Marcotrigiano J, Gingras AC, Sonenberg N, Burley SK (1997). Cocrystal structure of the messenger RNA 5’ cap-binding protein (elF4E) bound to 7-methyl-GDP. *Cell*.

[48] Matsuo H, Li H, McGuire AM (1997). Structure of translation factor elF4E bound to m7GDP and interaction with 4E-binding protein. *Nature Structural Biology*.

[49] Hershey PEC, McWhirter SM, Gross JD, Wagner G, Alber T, Sachs AB (1999). The cap-binding protein eIF4E promotes folding of a functional domain of yeast translation initiation factor eIF4G1. *Journal of Biological Chemistry*.

[50] Tomoo K, Shen X, Okabe K (2003). Structural features of human initiation factor 4E, studied by X-ray crystal analyses and molecular dynamics simulations. *Journal of Molecular Biology*.

[51] Altmann M, Muller PP, Pelletier J, Sonenberg N, Trachsel H (1989). A mammalian translation initiation factor can substitute for its yeast homologue in vivo. *Journal of Biological Chemistry*.

[52] Dhalia R, Reis CRS, Freire ER (2005). Translation initiation in Leishmania major: characterisation of multiple eIF4F subunit homologues. *Molecular and Biochemical Parasitology*.

[53] Lewdorowicz M, Yoffe Y, Zuberek J (2004). Chemical synthesis and binding activity of the trypanosomatid cap-4 structure. *RNA*.

[54] Freire ER, Dhalia R, Moura DMN (2011). The four trypanosomatid eIF4E homologues fall into two separate groups, with distinct features in primary sequence and biological properties. *Molecular and Biochemical Parasitology*.

[55] Yoffe Y, Léger M, Zinoviev A (2009). Evolutionary changes in the Leishmania eIF4F complex involve variations in the eIF4E-eIF4G interactions. *Nucleic Acids Research*.

[56] Zinoviev A, Leger M, Wagner G, Shapira M (2011). A novel 4E-interacting protein in Leishmania is involved in stage-specific translation pathways. *Nucleic Acids Research*.

[57] Hiremath LS, Hiremath ST, Rychlik W, Joshi S, Domier LL, Rhoads RE (1989). *in vitro* synthesis, phosphorylation, and localization on 48 S initiation complexes of human protein synthesis initiation factor 4E. *Journal of Biological Chemistry*.

[58] Rau M, Ohlmann T, Morley SJ, Pain VM (1996). A reevaluation of the Cap-binding protein, eIF4E, as a rate-limiting factor for initiation of translation in reticulocyte lysate. *Journal of Biological Chemistry*.

[59] Kramer S, Queiroz R, Ellis L (2008). Heat shock causes a decrease in polysomes and the appearance of stress granules in trypanosomes independently of eIF2*α* phosphorylation at Thr169. *Journal of Cell Science*.

[60] Cassola A, De Gaudenzi JG, Frasch AC (2007). Recruitment of mRNAs to cytoplasmic ribonucleoprotein granules in trypanosomes. *Molecular Microbiology*.

[61] Marcotrigiano J, Lomakin IB, Sonenberg N, Pestova TV, Hellen CUT, Burley SK (2001). A conserved HEAT domain within eIF4G directs assembly of the translation initiation machinery. *Molecular Cell*.

[62] Marintchev A, Wagner G (2005). eIF4G and CBP80 share a common origin and similar domain organization: implications for the structure and function of eIF4G. *Biochemistry*.

[63] Mader S, Lee H, Pause A, Sonenberg N (1995). The translation initiation factor eIF-4E binds to a common motif shared by the translation factor eIF-4*γ* and the translational repressors 4E-binding proteins. *Molecular and Cellular Biology*.

[64] Dhalia R, Marinsek N, Reis CRS (2006). The two eIF4A helicases in Trypanosoma brucei are functionally distinct. *Nucleic Acids Research*.

[65] Silva LMD, Owens KL, Murta SMF, Beverley SM (2009). Regulated expression of the Leishmania major surface virulence factor lipophosphoglycan using conditionally destabilized fusion proteins. *Proceedings of the National Academy of Sciences of the United States of America*.

[66] Linder P (2006). Dead-box proteins: a family affair–active and passive players in RNP-remodeling. *Nucleic Acids Research*.

[67] Ferraiuolo MA, Lee CS, Ler LW (2004). A nuclear translation-like factor eIF4AIII is recruited to the mRNA during splicing and functions in nonsense-mediated decay. *Proceedings of the National Academy of Sciences of the United States of America*.

[68] Kahvejian A, Svitkin YV, Sukarieh R, M’Boutchou MN, Sonenberg N (2005). Mammalian poly(A)-binding protein is a eukaryotic translation initiation factor, which acts via multiple mechanisms. *Genes and Development*.

[69] Deo RC, Bonanno JB, Sonenberg N, Burley SK (1999). Recognition of polyadenylate RNA by the poly(A)-binding protein. *Cell*.

[70] Wells SE, Hillner PE, Vale RD, Sachs AB (1998). Circularization of mRNA by eukaryotic translation initiation factors. *Molecular Cell*.

[71] Derry MC, Yanagiya A, Martineau Y, Sonenberg N (2006). Regulation of poly(A)-binding protein through PABP-interacting proteins. *Cold Spring Harbor Symposia on Quantitative Biology*.

[72] Jackson RJ (2005). Alternative mechanisms of initiating translation of mammalian mRNAs. *Biochemical Society Transactions*.

[73] da Costa Lima TD, Moura DMN, Reis CRS (2010). Functional characterization of three Leishmania poly(A) binding protein homologues with distinct binding properties to RNA and protein partners. *Eukaryotic Cell*.

[74] Bates EJ, Knuepfer E, Smith DF (2000). Poly(A)-binding protein I of Leishmania: functional analysis and localisation in trypanosomatid parasites. *Nucleic Acids Research*.

[75] Quijada L, Soto M, Alonso C, Requena JM (2000). Identification of a putative regulatory element in the 3′-untranslated region that controls expression of HSP70 in Leishmania infantum. *Molecular and Biochemical Parasitology*.

[76] Folgueira C, Quijada L, Soto M, Abanades DR, Alonso C, Requena JM (2005). The translational efficiencies of the two Leishmania infantum HSP70 mRNAs, differing in their 3′-untranslated regions, are affected by shifts in the temperature of growth through different mechanisms. *Journal of Biological Chemistry*.

[77] Zilka A, Garlapati S, Dahan E, Yaolsky V, Shapira M (2001). Developmental regulation of heat shock protein 83 in Leishmania: 3′ processing and mRNA stability control transcript abundance, and translation is directed by a determinant in the 3′-untranslated region. *Journal of Biological Chemistry*.

[78] Larreta R, Soto M, Quijada L (2004). The expression of HSP83 genes in Leishmania infantum is affected by temperature and by stage-differentiation and is regulated at the levels of mRNA stability and translation. *BMC Molecular Biology*.

[79] David M, Gabdank I, Ben-David M (2010). Preferential translation of Hsp83 in Leishmania requires a thermosensitive polypyrimidine-rich element in the 3′
UTR and involves scanning of the 5′ UTR. *RNA*.

[80] Markham NR, Zuker M (2008). UNAFold: software for nucleic acid folding and hybridization. *Methods in Molecular Biology*.

[81] Boucher N, Wu Y, Dumas C (2002). A common mechanism of stage-regulated gene expression in Leishmania mediated by a conserved 3′-untranslated region element. *Journal of Biological Chemistry*.

[82] McNicoll F, Müller M, Cloutier S (2005). Distinct 3′
-untranslated region elements regulate stage-specific mRNA accumulation and translation in Leishmania. *Journal of Biological Chemistry*.

[83] Moraes MCS, Jesus TCL, Hashimoto NN (2007). Novel membrane-bound eIF2*α* kinase in the flagellar pocket of Trypanosoma brucei. *Eukaryotic Cell*.

[84] Lahav T, Sivam D, Volpin H (2011). Multiple levels of gene regulation mediate differentiation of the intracellular pathogen Leishmania. *FASEB Journal*.

[85] Argaman M, Aly R, Shapira M (1994). Expression of heat shock protein 83 in Leishmania is regulated post-transcriptionally. *Molecular and Biochemical Parasitology*.

[86] Peacock CS, Seeger K, Harris D (2007). Comparative genomic analysis of three Leishmania species that cause diverse human disease. *Nature Genetics*.

[87] Haile S, Papadopoulou B (2007). Developmental regulation of gene expression in trypanosomatid parasitic protozoa. *Current Opinion in Microbiology*.

[88] Rosenzweig D, Smith D, Opperdoes F, Stern S, Olafson RW, Zilberstein D (2008). Retooling Leishmania metabolismml: from sand fly gut to human macrophage. *FASEB Journal*.

[89] Jaramillo M, Gomez MA, Larson O (2011). Translational control of Leishmania infection throught mTORC1 signaling. *Cell Host and Microbe*.

[90] Moerke NJ, Aktas H, Chen H (2007). Small-molecule inhibition of the interaction between the translation initiation factors eIF4E and eIF4G. *Cell*.

